# Risk Factors for Musculoskeletal Disorders in Korean Farmers: Survey on Occupational Diseases in 2020 and 2022

**DOI:** 10.3390/healthcare12202026

**Published:** 2024-10-11

**Authors:** Jinheum Kim, Kanwoo Youn, Jinwoo Park

**Affiliations:** 1 Department of Applied Statistics, University of Suwon, Hwaseong 18323, Republic of Korea; jkimdt65@gmail.com; 2 Department of Occupational & Environmental Medicine, Wonjin Green Hospital, Seoul 02221, Republic of Korea; dudunanum@hanmail.net; 3 Department of Data Science, University of Suwon, Hwaseong 18323, Republic of Korea

**Keywords:** AUC, class imbalance, model evaluation, prevalence, resampling

## Abstract

Background/Objectives: This study investigated factors influencing the prevalence of musculoskeletal disorders (MSDs) resulting from agricultural work, utilizing the 2020 and 2022 occupational disease survey data collected by the Rural Development Administration. The combined data from these years indicated a 6.02% prevalence of MSDs, reflecting a significant class imbalance in the binary response variables. This imbalance could lead to classifiers overlooking rare events, potentially inflating accuracy assessments. Methods: We evaluated five distinct models to compare their performance using both original and synthetic data and assessing the models’ performance based on synthetic data generation. In the multivariate logistic model, we focused on the main effects of the covariates as there were no statistically significant second-order interactions. Results: Focusing on the random over-sampling examples (ROSE) method, gender, age, and pesticide use were particularly impactful. The odds of experiencing MSDs were 1.29 times higher for females than males. The odds increased with age: 2.66 times higher for those aged 50–59, 4.60 times higher for those aged 60–69, and 7.16 times higher for those aged 70 or older, compared to those under 50. Pesticide use was associated with 1.26 times higher odds of developing MSDs. Among body part usage variables, all except wrists and knees were significant. Farmers who frequently used their necks, arms, and waist showed 1.27, 1.11, and 1.23 times higher odds of developing MSDs, respectively. Conclusions: The accuracy of the raw method was high, but the ROSE method outperformed it for precision and F1 score, and both methods showed similar AUC.

## 1. Introduction

To understand the extent of diseases and injuries caused by agricultural work among Korean farmers, and to develop an agricultural safety and health policy to improve farmers’ quality of life, the Rural Development Administration (RDA) initiated the “survey of occupational diseases and injuries of farmers” in 2009, designating it as a nationally approved statistic [1]. This survey, which alternates between disease and injury assessments, is conducted every other year. Selected farmers are visited directly and interviewed about diseases or injuries related to agricultural work that occurred in the past year. Since the survey relied on farmers’ recollections, it took the form of a recall survey. While research on farmers’ injuries is relatively active, both domestically and internationally, studies focusing on diseases are relatively rare. In South Korea, research on farmers’ diseases has typically been limited to specific conditions, particular types of agriculture, or specific regions, with comprehensive studies on farmers’ diseases being nearly non-existent.

A literature review was conducted on occupational diseases among farmers, which highlighted conditions that are more prevalent in agriculture than in other occupational groups [2]. Several studies focused on musculoskeletal disorders (MSDs), which represent the largest proportion of diseases among farmers [3,4,5,6,7,8]. In Poland, data on diseases of agricultural workers were analyzed by extracting information from Polish industrial accident records from 2000 to 2004 [9]. Due to the comprehensive registration of all patient information in the administrative system, research in Poland can leverage the full datasets obtained through administrative reports. Similarly, compensation claims for farm injuries and diseases were analyzed using time-series data from Finnish farmers insured by industrial accident insurance over 26 years (1982–2008) [10]. MSDs have emerged as the most common diseases related to agricultural work among Korean farmers. According to survey data, the prevalence of MSDs was 5.18% in 2020 and increased to 6.72% in 2022. Despite being the most frequently reported condition, the overall prevalence of MSDs in farming populations remains relatively low. This study aimed to identify the risk factors associated with MSDs in agricultural work and to develop basic guidelines aimed at reducing the incidence of MSDs among farmers.

However, a significant disparity between the incidence of events and non-events, such as the prevalence of MSDs among Korean farmers, can lead to an overestimation of the accuracy of classifiers [11]. This occurs because rare events are often overlooked and misclassified as more common during the model estimation stage for the training dataset. Studies have shown that logistic regression models underestimate the conditional probability of rare events [12]. Similarly, linear discriminant analysis (LDA) often relies on the dispersion of the prevalent class, as the common covariance matrix is estimated as a weighted average of the covariance matrices of each class [13]. Both parametric methods, such as logistic regression models and LDA, and more flexible non-parametric methods, such as decision trees and support vector machines, struggle with response variables that have a skewed distribution and optimize objective functions, such as accuracy [3,14]. To address the issue of imbalanced classification, focusing on the model estimation stage has been found to be an effective approach. In an attempt to mitigate this imbalance [15,16], techniques such as “under-sampling” and “over-sampling” have been proposed to create more balanced samples between the two classes [17]. Under-sampling involves extracting samples from each category without replacement so that the number of samples is the same as that of the least frequent category. The least frequent category remains unchanged, and under-sampling is only performed on the remaining categories. In contrast, over-sampling involves extracting samples using the replacement sampling method to maintain equal numbers of samples in each category. The original data remain unchanged, and over-sampling is only performed on the remaining categories, excluding the most frequent one. However, under-sampling may ignore useful samples, while over-sampling may lead to duplicated samples and overfitting [18]. To overcome these challenges, it has been suggested that noise be added with a normal distribution to rare event samples [19] and samples be randomly selected from the line segment connecting the nearest sample in feature space and the rare event sample, known as the synthetic minority over-sampling technique (SMOTE) [20]. The adaptive synthetic (ADASYN) sampling approach [21] dynamically adjusts the number of synthetic samples created based on the difficulty of classifying rare event samples. The more difficult a sample is to classify, the more synthetic samples are generated around it. This is determined by the density of the nearest neighbors of the rare event. A strategy to lessen the effect of class imbalance in a categorical response variable on model estimates and model evaluations was provided with the random over-sampling examples (ROSE) approach [11], which is based on the smoothing bootstrap methodology [22]. Unlike methods such as SMOTE or ADASYN, ROSE applies random perturbations to both rare and common event samples. This makes it a more general over-sampling technique that enhances the diversity of the data rather than focusing exclusively on rare event boundaries. Meanwhile, more recent variants such as borderline-SMOTE and K-means SMOTE have been developed to generate more sophisticated borderline samples around minority classes or to address noise issues [23]. More recently, research on loss functions and model structures has been conducted. Focal loss, which learns from unbalanced data by underweighting the prevalent event class and overweighting the rare event class, was the focus of [24]. In contrast, generative adversarial networks, which provide an effective method for learning features from rare event classes, were studied by [25].

In this study, due to the imbalance in the distribution of the response variables related to the prevalence of MSDs in agricultural work, a resampling method such as ROSE, which includes both under- and over-sampling, was employed to generate synthetic or augmented data. Among various statistical analysis approaches, a parametric additive logistic regression model was applied to identify the risk factors influencing the prevalence of MSDs among Korean farmers.

This remainder of this article is structured as follows. Section 2 provides a brief introduction to the ROSE algorithm [11] and the R package implementation [26], outlines the data collection procedure, and introduces the response variable and predictor variables used in this study. Section 3 presents the results of the association test between the presence of MSDs related to agricultural work and the predictor variables, along with the outcomes of the logistic regression model based on both original and synthetic data. Additionally, the sensitivity, precision, F_1_ score, and accuracy are compared across different threshold values to evaluate the logistic regression models. Finally, Section 4 offers guidelines for reducing the prevalence of MSDs among farmers, focusing on sociodemographic factors and the frequency of body part usage; it also discusses the limitations of this study.

## 2. Materials and Methods

### 2.1. Study Design and Settings

In this study, we used survey data on occupational diseases among farmers collected by the National Academy of Agricultural Sciences under the RDA in 2020 and 2022 (data source: https://kosis.kr/index/index.do, accessed on 25 April 2023). The dataset consisted of 14,075 participants in 2020 and 16,473 participants in 2022. After excluding 1205 individuals whose primary type of agriculture was not classified, 29,343 participants were selected for the analysis. To investigate the factors influencing the prevalence of MSDs among farmers, we considered study variables such as household members, farmers, and characteristics of exposure to risk factors.

Household characteristics included sex, age, duration of agricultural activity in the past year, and pesticide use. The past year refers to 2019 in the 2020 survey and 2021 in the 2022 survey. Age was categorized as “less than 50”, “50 to 59”, “60 to 69”, and “70 or older”, while the duration of farming activity was classified as “less than 6 months” or “6 months or more”. Pesticide use was defined as “yes” if pesticides were used directly or indirectly, and “no” otherwise. The farmers’ characteristics considered in this study included primary types of agriculture and household income. The types of agriculture were grouped into five categories: “rice”, “dry field”, “orchards”, “greenhouses”, and “livestock”. Household income was divided into four categories: “less than US$3800”, “US$3800–US$14,999”, “US$15,000–US$37,999”, and “US$38,000 or more”.

The characteristics of risk factor exposure included whether participants’ body parts were in uncomfortable postures while performing agricultural work. Specifically, this encompassed whether the neck was bent or hyper-extended, if the arms were raised above shoulder level, whether fingers or wrists were used repeatedly, whether there was a posture where the waist was bent or twisted to the side, and whether postures such as squatting or bending the knees were assumed. Additionally, we assessed whether participants engaged in work involving lifting objects weighing 10–19 kg or more than 20 kg. Responses of always and frequently were classified as “yes”, while responses of sometimes or rarely were classified as “no”. The outcome variable was defined as “yes” if the individual had experienced at least one MSD either caused or worsened by agricultural work over the past year and “no” if no such condition was reported.

### 2.2. Statistical Methods

#### 2.2.1. Review on the ROSE Resampling Strategy

$T_{n}$ was used as a training set consisting of n samples. Here, $T_{n}$ represented a collection of realizations for pairs $\left(Y,x\right)$ with Y as a categorical response variable and x as a vector of predictors ${\left(x_{1},x_{2},\ldots ,x_{d}\right)}^{\prime }$. Specifically, $T_{n}=\left\{\left(x_{i}^{\prime },y_{i}\right),\;i=1,2,\ldots ,n\right\}.$ Here, $x_{i}\in R^{d}$ followed an unknown probability density function *f*(***x***). For convenience, the category labels of Y were denoted with J labels $L_{1},\;L_{2},\ldots ,L_{J}.$ Additionally, $n_{j}$ (where $n_{j}<n$) represented the number of samples corresponding to the category label $L_{j}$ for j=1,2,…,J, such that $n=\sum_{j=1}^{J}n_{j}.$ The following steps were required to generate a synthetic training set:

Step 1: $y^{*}$ was selected with probability $\pi_{j}=\frac{1}{J}.\;$ Let $y^{*}=L_{j}.$

Step 2: One sample from the training dataset $T_{n}$ was randomly selected among individuals whose labels were $y^{*}$ with a probability of $p_{j}=\frac{1}{n_{j}}.$ That is, $\left(x_{i},y_{i}\right),$ where $y_{i}=y^{*}.$

Step 3: Synthetic samples were generated by using the smooth kernel estimation method around the feature space of the extracted training samples $\left(x_{i},y_{i}\right).$ Specifically, $\left(x^{*},y^{*}\right),$ where $x^{*}$ was randomly chosen from a normal distribution centered at $x_{i}$ with a covariance matrix $H_{j}$ (i.e., using a Gaussian kernel). At this stage, the smooth diagonal matrix was defined as $H_{j}=diag\left\{h_{1}^{\;(j)},h_{2}^{\;(j)},\ldots ,h_{d}^{\;(j)}\right\}$ with

$$h_{q}^{\;(j)}={\left(\frac{4}{\left(d+2\right)n}\right)}^{\left(\frac{1}{d+4}\right)}{\hat{\sigma }}_{q}^{\;(j)},\;q=1,2,\ldots ,d,$$

where ${\hat{\sigma }}_{q}^{\;(j)}$ represented the sample standard deviation of the q-th predictor of training samples belonging to the class label $L_{j}$ [27].

Step 4: The process outlined in Steps 1–3 was iteratively performed until there was a nearly equivalent number of synthetic samples in each category and the overall count of synthetic samples closely matched the number of samples in $T_{n}.$ Finally, we set $T_{m}^{*}$ representing the synthetic training set.

For model evaluation, the R package ROSE integrated the leave-K-out cross-validation (LKOCV) and bootstrap methods, replacing the resubstitution and validation set methods. The resubstitution method may lead to an overestimation of the model accuracy, while the validation set method may exhibit high variability due to random division. The LKOCV method was preferred because, similar to the validation set method, it involved random division but showed less variability. However, the LKOCV method required a longer computation time as it involves repeated model estimation, unlike the validation set or holdout methods.

Step 1: The training dataset $T_{n}$ was randomly divided into $Q=\frac{n}{K}$ folds, denoted as $T_{K}^{1},\;T_{K}^{2},\ldots ,T_{K}^{Q},$ where each fold contained K samples. In cases where n was not a multiple of K, $\left(Q-1\right)$ folds had the same number of samples, and the remaining fold consisted of the leftover samples.

Step 2: One fold out of the Q folds (denoted as $T_{K}^{i}$) was chosen and the remaining $\left(Q-1\right)$ folds were grouped (denoted as ${T_{n}^{-i}=T}_{n}-T_{K}^{i}$).

Step 3: The ROSE method was used by setting $T_{n}^{-i}$ as the training set and a synthetic training set from $T_{n}^{-i}$ (denoted as $T_{m}^{-i}$) was generated according to the procedure described in Section 2.1.

Step 4: The model was estimated using the synthetic training set $T_{m}^{-i}$ and predictions for the samples in the holdout set $T_{K}^{i}$ were obtained (denoted as $P_{K}^{i}$).

Step 5: For each of the Q folds, Steps 2 through 4 were repeated to generate predictions for the samples in each fold (i.e., $P_{K}^{1},P_{K}^{2},\ldots ,P_{K}^{Q}).$ The accuracy was calculated based on these predictions.

Therefore, the accuracy of the LKOCV method was defined as:

$$\frac{1}{n}\sum_{q=1}^{Q}\sum_{j\in T_{K}^{q}}I\;(y_{j}=P_{K,j}^{q}),$$

where $P_{K,j}^{q}$ represented the predicted value for the j-th sample in the set $T_{K}^{q}\;\left(q=1,2,\ldots ,Q;\;j=1,2,\ldots ,K\right).$

While the error rate (or accuracy) is a commonly used measure for model evaluation, it has limitations, particularly in the context of imbalanced data. In such cases, models may exhibit a bias towards the majority class, leading to an overestimation of accuracy (or an underestimation of the error rate) [28]. For example, in a dataset with a rare event rate of 1%, a “naive” classifier that predicts all samples as the majority class would yield an error rate of just 1%, thus underestimating the true error rate.

This underscores the importance of selecting appropriate evaluation metrics when dealing with unbalanced data. Unlike the error rate, which emphasizes false positives and false negatives as shown in the confusion matrix, metrics such as precision and recall focus on true positives. Precision is defined as the proportion of true positives among all samples predicted as positive, while recall (or sensitivity) is the proportion of actual positive samples correctly identified.

In addition, the F_1_ score, which is the harmonic mean of precision and recall, is often used to provide a balanced measure of a model’s performance [29]. Another widely used metric is the receiver operating characteristic (ROC) curve, which plots the true-positive rate against the false-positive rate at various threshold settings. The area under the curve (AUC) represents the area under the ROC curve, with values closer to 1 indicating a better-performing model. These measures, particularly in the context of imbalanced data, offer a more profound understanding of model performance than simple accuracy or error rates.

#### 2.2.2. Statistical Analysis

Pearson’s large-sample chi-square test was used to determine if the study variables were marginally associated with MSDs. To further investigate factors influencing the prevalence of MSDs, a multivariate logistic regression analysis was conducted. Given the imbalance in the dataset, where the number of subjects with MSDs was significantly lower than those without, resampling methods [11,26] were employed. These methods generated synthetic or artificial samples, balancing the number of subjects with and without MSDs, followed by logistic regression analysis on these balanced samples. The performance of the proposed regression models was evaluated using various metrics, such as Nagelkerke’s R-squared statistic [30], precision, recall, F_1_ score, accuracy, and AUC. These metrics offer a comprehensive comparison of the models’ predictive capabilities regarding the prevalence of MSDs.

## 3. Results

Data of the contingency and association tests between each study variable and MSDs are shown in Table 1. MSDs are rare occurrences, with an estimated prevalence rate of 6.02%. The incidence rate was strongly associated with sex, age, and household income. With the exception of the wrist, the neck, arms, waist, and knees were substantially associated with MSDs among the body parts exposed to risks during agricultural work. The task of lifting objects weighing 10 to 19 kg or over 20 kg was closely correlated with the presence of MSDs. The duration of farming activity, type of farming, and pesticide use showed a slight relationship with MSDs.

Table 2 presents the point estimates and 95% confidence intervals (CIs) for the odds ratios related to the prevalence of MSDs, while Table 3 presents the estimated performance measures for various models. In this study, we evaluated five distinct models to assess their comparative performance using both original and synthetic data. We also examined the performance of the models based on the method of synthetic data generation. In the multivariate logistic model, we focused on the main effects of the covariates, as there were no statistically significant second-order interactions. The “raw” column shows the regression coefficients derived from the original dataset, which included 29,343 individuals (1765 with and 27,578 without MSDs). In contrast, the “synthetic” column encompasses the results from four resampling methods: “under”, “over”, “both”, and “ROSE”, which were applied to generate balanced datasets with nearly equal numbers of individuals with and without MSDs.

In the “under” method, the number of individuals without MSDs was reduced to match those with MSDs (3529 individuals: 1765 with and 1764 without MSDs). The “over” method increased the number of individuals without MSDs to match the number of those with MSDs, resulting in 55,263 individuals (27,685 with and 27,578 without MSDs). The “both” and “ROSE” methods involved under- and over-sampling, resulting in 29,343 synthetic individuals each. For the “both” and “ROSE” methods, the number of samples from each class (with and without MSDs) was set to be equal, with sampling probabilities set to 0.5, to optimize model performance. This was evidenced by the higher precision, recall, and AUC in unreported results.

**Figure 1:**
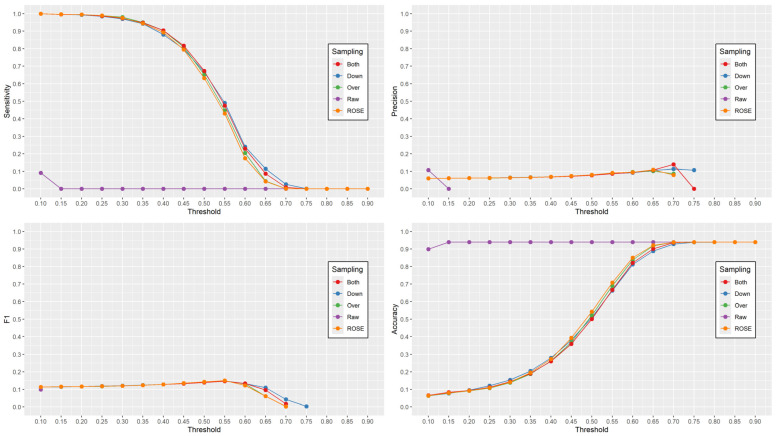
Sensitivity, precision, F1 score, and accuracy plots based on raw and synthetic samples.

**Figure 2:**
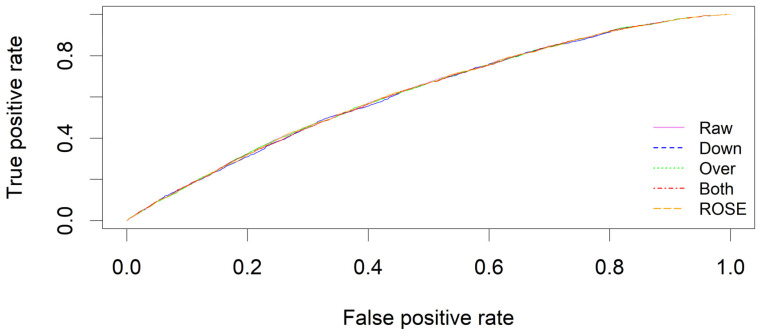
Receiver operating characteristic curves based on raw and synthetic samples.

Goodness-of-fit measures were calculated by applying the models to the entire set of 29,343 individuals, using a threshold value of 0.5 for classification. Figure 1 displays the recall (sensitivity), precision, F_1_ score, and accuracy of each model at different thresholds, increasing by 0.05 from 0.1 to 0.9. The ROC curves for the five models indicated that the AUCs were similar across the models (Figure 2 and Table 3).

The models, generated using synthetic data, were comparable across all four performance measures. However, despite demonstrating high accuracy, the raw model had a recall of 0 and an undefined precision and F_1_ score. The estimated probability of MSDs in the raw model for each of the 29,343 individuals was lower than the threshold value of 0.5, resulting in all individuals being classified as not having MSD. This highlights the misleading nature of the high accuracy of the raw model, which stems from the fact that the majority group (without MSDs) was correctly classified, but no cases of MSD were identified.

The models in the synthetic column outperformed the raw model for Nagelkerke’s R-squared statistic, which was approximately two times higher. Among the resampling methods, no significant difference was observed in model performance. However, the “under” sampling method yielded the highest Nagelkerke’s R-squared statistic value. The “over” and “both” sampling methods achieved the highest sensitivity, while the “both” sampling method showed the highest precision and F_1_ score. Interestingly, the “ROSE” method demonstrated the highest accuracy. Most variables were highly significant (*p*-value = 0.01) in the ROSE column, except for the household income (*p*-value = 0.077), wrist (*p*-value = 0.039), and knee (*p*-value = 0.159). Specifically, females showed 1.29 times higher odds of experiencing MSDs compared to males. The odds increased with age: 2.66 times higher for those aged 50–59, 4.60 times higher for those aged 60–69, and 7.16 times higher for those aged 70 or older, compared to those under 50. Farmers with less than 6 months of agricultural activities had 1.25 times greater odds of developing MSDs than those with 6 months or more. Pesticide use was associated with 1.26 times higher odds of developing MSDs.

For different farming types, the odds of MSD occurrence were 1.15 times higher for greenhouse farming than for rice farming. However, livestock, dry field, and orchard farming were associated with 0.97, 0.90, and 0.75 times lower odds of MSDs, respectively. Households with incomes ranging from USD 3800 to USD 14,999 and USD 15,000 to USD 37,999 had 0.96 and 0.92 times lower odds of experiencing MSDs, respectively, compared to households with incomes less than USD 3800. Finally, farmers who frequently used their neck, arms, and waists demonstrated 1.27, 1.11, and 1.23 times higher odds of developing MSDs, respectively. Lifting objects weighing more than 20 kg was associated with 1.14 times higher odds of developing MSDs, while lifting objects of 10–19 kg was, paradoxically, associated with 1.19 times higher odds of MSDs for those who rarely lifted such objects compared to those who frequently lifted.

## 4. Discussion

In this study, we analyzed the factors influencing the prevalence of MSDs among agricultural workers, using data from the 2020 and 2022 occupational disease surveys conducted by the RDA. The combined data from 2020 and 2022 revealed a 6.02% prevalence of MSDs associated with agricultural work, indicating a significant class imbalance in the binary response variables. To address this class imbalance, we applied resampling methods [11,26] to improve the accuracy of our model estimation. The following trends were identified by analyzing the prevalence of MSDs in farmers without considering other risk factors. Farmers in their 60 s and 70 s exhibited prevalence rates that were 3.8% and 6.3% higher, respectively, than those in their 50 s, with a steady increase in prevalence with age. This finding has been commonly observed in research related to MSDs in agricultural work [8,31]. The mechanism of MSDs associated with agricultural work is attributed to the exposure to cumulative ergonomic risk factors. Aging exacerbates this phenomenon by increasing the duration of exposure and decreasing the body’s ability, thereby leading to a higher prevalence of MSDs [32]. Farmers routinely work beyond the standard retirement age, often farming at the age of 70 or more. MSDs related to work exposure are enhanced by age-related musculoskeletal changes, increasing the chances of having high-severity MSDs [33].

The prevalence of MSDs was 2% higher in women than in men, primarily because of their physical sensitivity to ergonomic risk factors, such as handling heavy loads and the repetitive nature of work [34]. Beyond physical conditions, socioeconomic characteristics such as lower household income and single-person households contribute to the reduced use of agricultural machinery, lower healthcare access, and an increased burden of domestic chores. These factors further increase the risk of MSDs [35]. Although the MSD prevalence did not differ significantly by farming types, it was higher among those engaged in rice paddy, field, or greenhouse farming than those involved in livestock or orchard farming. There was a little variation in the level of exposure to ergonomic risk factors across farming types [36]. Additionally, because many agricultural workers engaged in multiple types of crops, there was a minimal difference in the prevalence of MSDs by the type of farming. However, in livestock and orchards, the relatively younger age of agricultural workers and greater use of agricultural machinery contributed to a comparatively lower prevalence of MSDs.

Farmers earning less than USD 3800 had a 2.5% higher prevalence rate of MSDs compared to those with incomes exceeding USD 38,000. To prevent MSDs, it is essential to use agricultural machinery and convenient equipment to reduce exposure to ergonomic risk factors. However, in low-income countries, making preventative investments is difficult. Additionally, limited income restricts access to healthcare services, impeding early management of MSDs. Furthermore, limitations in agricultural work performance due to MSDs lead to reduced productivity, creating a vicious cycle in which decreased income results in fewer resources for prevention and management [37].

The waist showed the most significant difference in prevalence rates among body parts. Farmers who frequently bent or twisted their waists had a 1.5% higher prevalence of MSDs compared to those who did not. The prevalence of low back pain (LBP) among agricultural workers is approximately 50%, significantly higher than that among industrial workers, who have a prevalence of approximately 37% [38]. The annual incidence of chronic LBP (lasting more than three months) is approximately 10% [39]. The prevalence of musculoskeletal pain among Korean farmers is exceptionally high at 97.2%, with LBP accounting for 58.7% of the cases [40]. Various occupational risk factors contribute to low back disorders, key risk factors of which include handling heavy loads and maintaining a bent back posture during work [41]. Despite advancements in agricultural mechanization in South Korea, many agricultural tasks are still performed manually. In rice paddies, fields, and greenhouse farming, the height of crops is typically below waist level, leading to most tasks being performed in a bent-over posture. Additionally, the lack of lightweight agricultural materials results in frequent handling of heavy items, contributing to the high prevalence of MSDs in the lumbar region [42,43].

Compared to those who rarely lifted heavy objects, the prevalence rate of MSDs was 1.5% higher among farmers who regularly lifted objects weighing less than 20 kg, and 0.9% higher for those lifting objects weighing more than 20 kg. The incidence of MSDs in the lumbar region among agricultural workers increased significantly when regularly lifting weights of approximately 20 kg or higher [44]. Many tasks performed by agricultural workers involve postures that can induce MSDs in the lumbar region, with lifting heavy objects identified as the most hazardous posture [45].

Agriculture in South Korea is dominated by small-scale self-employed farmers. Unlike other industries, the agricultural sector has been slow to adopt automation and mechanization. Additionally, the rural population in South Korea is aging at a rate faster than the urban population [46]. These socioeconomic characteristics likely contribute to the risk factors for MSDs in Korean farmers. Similar trends have been observed in studies of MSDs in Southeast Asian farmers, who share similar agricultural characteristics with Korea. A systematic review of MSD studies in 11 Southeast Asian countries revealed common risk factors, including increasing age, female gender, and heavy lifting [3]. A meta-analysis of 64 MSD studies from 23 low- and middle-income countries found that lumbar spine disorders were the most common, with heavy lifting being a major risk factor [47].

To prevent MSDs, measures should be taken to address risk factors. Currently, a national pilot program in South Korea provides specialized health screenings for some female farmers that include musculoskeletal disease screening. This program should be expanded to include all women farmers and be linked not only to screening but also to education and early treatment for MSDs. Social security for low-income farmers should be strengthened. Reductions in insurance fees for low-income farmers are needed for agricultural workers’ compensation insurance, and coverage for MSDs should be strengthened. Additionally, reducing the volume and weight of fertilizers, pesticides, and other materials used during farming operations and agricultural products to less than 10 kg can help reduce the burden of heavy lifting. The development of agricultural machinery and material handling equipment that can be easily used by older farmers should be prioritized, especially in paddy, field, and greenhouse farming.

Among the resampling methods used to address class imbalance, the over-sampling method resulted in the largest sample size, while the under-sampling method had the smallest sample size. Despite the differences in the training samples, the model evaluation metrics remained consistent across the methods. However, the ROSE method demonstrated significantly different performance compared to the raw method. The raw method classified all samples as “no MSDs”, resulting in undefined precision and F_1_ score. Although the raw method had higher accuracy, the ROSE method outperformed it for recall, precision, and F_1_ scores, with both methods showing similar AUC.

In the ROSE method, the key sociodemographic variables, such as gender, age, farming type, household income, farming activity period, and pesticide use, were statistically significant. Among these factors, sex, age, and pesticide use were particularly important. The prevalence of MSD was higher among women, farmers who used pesticides (directly or indirectly), and older age groups (in descending order: 70 s, 60 s, 50 s, and <50 s). Among the body part usage variables, all except the wrists and knees were significant, with the neck, waist, and lifting objects weighing less than 20 kg being highly significant. The generalized variance inflation factor (GVIF) was calculated to ascertain the presence of multicollinearity among the covariates [48]. The GVIF values for all variables, including age, were approximately 1, indicating the absence of collinearity among the covariates and a consistency in the GVIF values across the five evaluated models.

Various methods, including resubstitution, validation set (holdout), cross-validation (CV), and bootstrapping, were considered for model evaluation. The bootstrap method is the most computationally intensive, involving the repeated estimation of models B times (for example, B = 100), while the CV method repeats as many times as the number of folds. Other methods estimate models only once. In this study, the resubstitution method was applied. In Step 2, as outlined in Section 2.1, selection probabilities for each class were specified through a grid search, with model performance measures determined by the optimal value. Since this survey was conducted in a 1:1 face-to-face manner, with an interviewer visiting the farmers, there was a no option for non-response. When unit non-response occurred, a replacement was made during follow-up. Additionally, incorrect responses were filtered out through logical editing before analysis, and values were replaced by nearest neighbor imputation. However, the impact of imputation was statistically negligible. The survey for this study was conducted twice: first in 2020 and then again in 2022, during the peak of the global COVID-19 pandemic. As a result, additional efforts were necessary, including establishment of safety protocols for field surveys and the provision of safety gear. In the 2022 survey, COVID-19 infection was also included as a separate category of disease type. Of 16,473 respondents in 2022, 1005 contracted COVID-19 and experienced symptoms, but none attributed it to farming. Therefore, it is challenging to determine if COVID-19 directly impacted agricultural diseases.

This study has limitations. First, it is a recall survey that relies on respondents’ memories. In Korea, there is a high proportion of elderly farmers, who primarily guessed their responses when asked to remember the situation of the past year. In some cases, they were not sure about the year of the event. This may have had a slight effect on the accuracy of their responses. To obtain more accurate and less biased information on healthcare utilization behaviors, future research should consider integrating data from the Health Insurance Review & Assessment Service database, rather than solely depending on the participants’ recall data. Second, the inherent sampling error in sample surveys is a crucial limitation. The survey was conducted on 16,473 out of approximately 2.3 million farmers in Korea. Even after excluding non-sampling errors, there remains inherent sampling error. Considering that the prevalence of MSDs caused by agricultural work is around 4%, the sample size of 16,473 is not optimal. However, due to budget constraints, increasing the sample size was challenging. It is important to acknowledge the presence of sampling error in the findings. Lastly, the models proposed in this study were not obtained from 100% real survey data; rather, they were from data obtained using resampling methods that expanded or reduced existing data. To overcome the significant decrease in model sensitivity when using a model based only on survey data, synthetic resampling methods were introduced. Therefore, it is necessary to recognize this limitation and interpret the results accordingly.

## 5. Conclusions

The accuracy of the raw method was high, but the ROSE method outperformed it for precision and F1 score, and both methods showed similar AUC.

## Figures and Tables

**Table 1:** Association test between each predictor and disease prevalence caused by musculoskeletal disorders.

		MSDs’ prevalence		
		No	Yes	
Predictor	Category	*n* (%)	*n* (%)	*p*-value
Total	-	27,578 (94.0)	1765 (6.0)	-
Sex	Male (ref)	13,623 (95.0)	718 (5.0)	<0.0001
	Female	13,955 (93.0)	1047 (7.0)	
Age, yrs	<50 (ref)	903 (98.9)	10 (1.1)	<0.00001
	50–59	2820 (97.2)	80 (2.8)	
	60–69-	7760 (95.1)	398 (4.9)	
	≥70	16,095 (92.6)	1277 (7.4)	
FAP, months	0–5 (ref)	1822 (93.1)	135 (6.9)	0.0889
	6–12	25,756 (94.0)	1630 (6.0)	
Pesticide	No (ref)	4491 (94.6)	254 (5.4)	0.0362
	Yes	23,087 (93.9)	1511 (6.1)	
Types of farming	Rice (ref)	10,521 (93.6)	784 (6.4)	0.0921
	Dry field	12,126 (94.1)	763 (5.9)	
	Orchard	2587 (94.9)	139 (5.1)	
	Greenhouse	1063 (94.2)	65 (5.8)	
	Livestock	281 (95.2)	14 (4.8)	
Income, US dollars	<3799 (ref)	9329 (93.1)	690 (6.9)	<0.0001
	3800–14,999	10,568 (93.9)	680 (6.1)	
	15,000–37,999	5656 (94.9)	302 (5.1)	
	≥38,000	2025 (95.6)	93 (4.4)	
Neck	No (ref)	13,324 (94.5)	770 (5.5)	0.0001
	Yes	14,254 (93.5)	995 (6.5)	
Arms	No (ref)	15,924 (94.4)	952 (5.6)	0.0017
	Yes	11,654 (93.5)	813 (6.5)	
Wrists	No (ref)	9649 (94.2)	589 (5.8)	0.1671
	Yes	17,929 (93.8)	1176 (6.2)	
Waist	No (ref)	5631 (95.3)	279 (4.7)	<0.0001
	Yes	21,947 (93.7)	1486 (6.3)	
Knees	No (ref)	6710 (94.9)	361 (5.1)	0.0003
	Yes	20,868 (93.7)	1403 (6.3)	
Lifting: 10–19, kg	No (ref)	15,091 (93.3)	1086 (6.7)	<0.0001
	Yes	12,487 (94.8)	679 (5.2)	
Lifting: ≥20, kg	No (ref)	18.752 (93.7)	1265 (6.3)	0.0013
	Yes	8826 (94.6)	500 (5.4)	

FAP: Farming activity period (unit: months).

**Table 2:** Odds ratio with the 95% confidence interval of each predictor on disease prevalence caused by musculoskeletal disorders from multivariable logistic regression analysis based on raw and synthetic samples.

Predictor	Category	Raw		Synthetic							
				Under		Over		Both		ROSE	
		OR (95% CI)	*p*-Value	OR (95% CI)	*p*-Value	OR (95% CI)	*p*-Value	OR (95% CI)	*p*-Value	OR (95% CI)	*p*-Value
Sex	Female	1.31 (1.17, 1.45)	<0.0001	1.42 (1.22, 1.64)	<0.0001	1.42 (1.22, 1.64)	<0.0001	1.42 (1.22, 1.64)	<0.0001	1.32 (1.26, 1.39)	<0.0001
Age, yrs Ref: <50	50–59	2.51 (1.30, 4.87)	<0.0001	1.93 (0.92, 4.06)	<0.0001	1.93 (0.92, 4.06)	<0.0001	1.93 (0.92, 4.06)	<0.0001	2.63 (2.05, 3.38)	<0.0001
	60–69	4.43 (2.35, 8.33)		3.40 (1.68, 6.88)		3.40 (1.68, 6.88)		3.40 (1.68, 6.88)		4.55 (3.59, 5.77)	
	≥70	6.64 (3.54, 12.5)		4.69 (2.32, 9.45)		4.69 (2.32, 9.45)		4.69 (2.32, 9.45)		6.69 (5.28, 8.47)	
FAP Ref: 0–5	6–12	0.79 (0.66, 0.95)	0.0151	0.84 (0.64, 1.09)	0.1923	0.84 (0.64, 1.09)	0.1923	0.84 (0.64, 1.09)	0.1923	0.88 (0.80, 0.96)	0.0053
Pesticide	Yes	1.24 (1.08, 1.43)	0.0020	1.27 (1.04, 1.54)	0.0165	1.27 (1.04, 1.54)	0.0165	1.27 (1.04, 1.54)	0.0165	1.23 (1.15, 1.32)	<0.0001
Types of farming	Dry field	0.88 (0.79, 0.98)	0.0429	0.78 (0.67, 0.90)	0.0208	0.78 (0.67, 0.90)	0.0208	0.78 (0.67, 0.90)	0.0208	0.86 (0.81, 0.90)	<0.0001
	Orchard	0.83 (0.68, 1.01)		0.87 (0.66, 1.15)		0.87 (0.66, 1.15)		0.87 (0.66, 1.15)		0.80 (0.73, 0.88)	
	Greenhouse	1.12 (0.86, 1.46)		0.93 (0.65, 1.34)		0.93 (0.65, 1.34)		0.93 (0.65, 1.34)		1.12 (0.99, 1.28)	
	Livestock	1.16 (0.67, 2.01)		1.25 (0.59, 2.68)		1.25 (0.59, 2.68)		1.25 (0.59, 2.68)		1.06 (0.81, 1.38)	
Income, US dollars Ref: <3800	3800–14,999	0.93 (0.83, 1.04)	0.4436	1.05 (0.89, 1.23)	0.8459	1.05 (0.89, 1.23)	0.8459	1.05 (0.89, 1.23)	0.8459	0.90 (0.86, 0.96)	0.0010
	15,000–37,999	0.90 (0.77, 1.04)		0.96 (0.78, 1.18)		0.96 (0.78, 1.18)		0.96 (0.78, 1.18)		0.92 (0.85, 0.99)	
	≥38,000	0.90 (0.71, 1.14)		1.00 (0.73, 1.38)		1.00 (0.73, 1.38)		1.00 (0.73, 1.38)		0.85 (0.76, 0.95)	
Neck	Yes	1.22 (1.08, 1.38)	0.0015	1.19 (1.00, 1.42)	0.0447	1.19 (1.00, 1.42)	0.0447	1.19 (1.00, 1.42)	0.0447	1.20 (1.13, 1.28)	<0.0001
Arms	Yes	1.14 (1.01, 1.29)	0.0336	1.11 (0.94, 1.32)	0.2219	1.11 (0.94, 1.32)	0.2219	1.11 (0.94, 1.32)	0.2219	1.19 (1.12, 1.26)	<0.0001
Wrists	Yes	0.93 (0.82, 1.06)	0.2877	0.95 (0.80, 1.15)	0.6204	0.95 (0.80, 1.15)	0.6204	0.95 (0.80, 1.15)	0.6204	0.94 (0.89, 1.00)	0.0642
Waist	Yes	1.26 (1.07, 1.49)	0.0046	1.42 (1.22, 1.64)	0.1529	1.42 (1.22, 1.64)	0.1529	1.42 (1.22, 1.64)	0.1529	1.27 (1.18, 1.38)	<0.0001
Knees	Yes	1.05 (0.91, 1.22)	0.4906	1.93 (0.92, 4.06)	0.2692	1.93 (0.92, 4.06)	0.2692	1.93 (0.92, 4.06)	0.2692	1.05 (0.98, 1.13)	0.1657
Lifting: 10–19 kg	Yes	0.78 (0.68, 0.90)	0.0006	3.40 (1.68, 6.88)	0.0022	3.40 (1.68, 6.88)	0.0022	3.40 (1.68, 6.88)	0.0022	0.74 (0.69, 0.79)	<0.0001
Lifting: ≥20 kg	Yes	1.15 (0.98, 1.34)	0.0828	4.69 (2.32, 9.45)	0.4183	4.69 (2.32, 9.45)	0.4183	4.69 (2.32, 9.45)	0.4183	1.17 (1.09, 1.26)	<0.0001

OR: odds ratio; CI: confidence interval; FAP: farming activity period; Under: under-sampling; Over: over-sampling; Both: under- and over-sampling; ROSE: random over-sampling examples.

**Table 3:** Measures of model performance from multivariable logistic regression analysis based on raw and synthetic samples.

		OR (95% CI)	*p*-Value	OR (95% CI)	*p*-Value	OR (95% CI)	*p*-Value	OR (95% CI)	*p*-Value	OR (95% CI)	*p*-Value
Measures	Nagelkerke’s R-square	0.029		0.075		0.064		0.065		0.074	
	Sensitivity	0		0.679		0.657		0.659		0.671	
	Precision	-		0.077		0.080		0.079		0.079	
	F_1_ score	-		0.069		0.071		0.071		0.070	
	Accuracy	0.940		0.508		0.524		0.518		0.508	
	AUC	0.619		0.615		0.618		0.615		0.618	

OR: odds ratio; CI: confidence interval; FAP: farming activity period; Under: under-sampling; Over: over-sampling; Both: under- and over-sampling; ROSE: random over-sampling examples; AUC: area under curve.

## Data Availability

The raw data supporting the conclusions of this article can be downloaded from https://kosis.kr/index/index.do (accessed on 25 April 2023).
